# Obstructive Sleep Apnea and Cardiovascular Diseases: A Systematic Review and Meta-Analysis of Prospective Studies

**DOI:** 10.7759/cureus.71752

**Published:** 2024-10-18

**Authors:** Shwan Amen, Banan Rasool, Bareq S Al Lami, Christiena Gamal Shehata, Aya N Mohammad, Payam Maaroof, Ramyar M Abdullah, Rasish Subedi, Raghad Al-Lami

**Affiliations:** 1 Cardiac Center, Surgical Specialty Hospital, Erbil, IRQ; 2 Research and Development, Erbil Cardiovascular Research Center, Erbil, IRQ; 3 Internal Medicine, Erbil Teaching Hospital, Erbil, IRQ; 4 College of Medicine, Hawler Medical University, Erbil, IRQ; 5 College of Health Sciences, Hawler Medical University, Erbil, IRQ; 6 Internal Medicine, Aiken Regional Medical Center, Aiken, USA; 7 Medicine, Universal College of Medical Sciences and Teaching Hospital, Bhairahawa, NPL; 8 Science, International School of Choueifat, Erbil, IRQ

**Keywords:** cardiovascular disease, cpap therapy, meta-analysis, obstructive sleep apnea, risk factors

## Abstract

Obstructive sleep apnea (OSA) is a prevalent clinical disorder characterized by intermittent airway obstruction during sleep, resulting in hypoxemia and hypercapnia. Although OSA is associated with increased cardiovascular disease (CVD) risk, such as hypertension, the precise nature of this relationship remains uncertain. This systematic review and meta-analysis aim to evaluate the potential links between OSA and cardiovascular outcomes by synthesizing data from recent prospective studies.

A systematic literature search was conducted following the Preferred Reporting Items for Systematic Reviews and Meta-Analyses (PRISMA) guidelines, utilizing electronic databases, including PubMed/MEDLINE, Scopus, and the Cochrane Library. The PICTS (patients, index test, comparator/reference test, target condition, study design) framework was used to structure the primary research question and define the investigation's scope. The Newcastle-Ottawa Scale (NOS) was employed for quality appraisal, and a meta-analysis was performed using Review Manager 5.4 software (Cochrane Collaboration, London, UK). The analysis included 12 studies, focusing on the association between OSA and various cardiovascular outcomes, including hypertension, coronary artery disease, congestive heart failure, cardiac arrhythmias, and cardiovascular events. The pooled relative risk (RR) from the random-effects model was 0.79 (95% CI: 0.56-1.03), indicating a non-significant reduction in cardiovascular risk associated with OSA. The results were heterogeneous, with individual studies showing both increased and decreased risk. Subgroup analyses based on study design, patient characteristics, and follow-up duration suggested that the observed associations were stable across different subsets of studies. However, the overall findings did not establish a definitive causal link between OSA and increased cardiovascular risk.

This meta-analysis underscores the complex relationship between OSA and CVD, highlighting the need for further research to elucidate the underlying mechanisms and confirm the potential causal association. Despite the lack of significant findings, the high prevalence of OSA and its association with cardiovascular risk factors warrant routine screening and early intervention, particularly through continuous positive airway pressure (CPAP) therapy, which may mitigate the cardiovascular risks linked to OSA. Future studies should focus on high-quality prospective data and explore the impact of OSA management on cardiovascular outcomes.

## Introduction and background

Obstructive sleep apnea (OSA) is a clinical disorder characterized by intermittent airway obstruction during sleep, leading to hypoxemia (an abnormally low concentration of oxygen in the blood) and hypercapnia (abnormally elevated carbon dioxide levels in the blood) [1]. OSA's prevalence is striking, affecting approximately 22% of men and 17% of women [2]. The condition's impact extends beyond sleep disturbances, with a notable association with various cardiovascular diseases (CVDs), including hypertension, coronary artery disease, congestive heart failure, pulmonary hypertension, cardiac arrhythmias, and more specifically, atrial fibrillation [3].

Mechanistically, the link between OSA and CVDs can be attributed to the hypoxemia resulting from airway obstruction, which triggers sympathetic activation, leading to elevated blood pressure and heart rate. Additionally, systemic inflammation, endothelial damage, and increased insulin resistance further contribute to cardiovascular pathology [4]. Of particular note, OSA is strongly correlated with hypertension and is recognized as one of the underlying causes of resistant hypertension [5]. Moreover, obesity, a well-known risk factor for coronary artery disease, exacerbates the likelihood of developing OSA [6,7]. Despite these established associations, conclusive evidence categorizing OSA as a direct risk factor for CVD remains elusive [8].

Furthermore, OSA is associated with numerous non-cardiac consequences, including cognitive impairment, daytime sleepiness, metabolic dysfunction, and an increased risk of road traffic accidents and mortality [9]. These complications significantly impact the quality of life and can lead to serious health issues if left untreated. Despite its high prevalence, OSA often remains undiagnosed, particularly among individuals with pre-existing cardiovascular conditions, who may not recognize the symptoms or may attribute them to other underlying health problems [10]. Although continuous positive airway pressure (CPAP) is widely regarded as the primary mode of treatment for OSA and is effective in alleviating symptoms such as daytime sleepiness and improved oxygenation during sleep [11], ongoing debates exist regarding its long-term effectiveness in reducing the risk of CVDs. Some studies suggest that CPAP therapy may lower blood pressure and improve cardiovascular outcomes, while others indicate that the impact on reducing cardiovascular risk remains inconclusive [12].

This study aims to conduct a comprehensive search, incorporating findings from prospective studies. The objective of this systematic review is to conduct a comprehensive analysis of recent prospective studies to better understand the association between OSA and CVDs. Specifically, the review aims to evaluate the potential links between OSA and cardiovascular outcomes. Through this analysis, the review seeks to provide evidence-based insights that can inform and enhance clinical practice.

## Review

Methods

Systematic Literature Search

This study followed the guidelines outlined in the Preferred Reporting Items for Systematic Reviews and Meta-analyses (PRISMA) statement [13]. The research methodology was structured around the PICTS (patients, index test, comparator/reference test, target condition, study design) framework to formulate the primary research question and define the scope of the investigation [14].

Search Strategy

A comprehensive literature search was conducted using electronic databases, including PubMed/MEDLINE, Google Scholar, and the Cochrane Library. Medical Subject Headings (MeSH) terms and Boolean operators were employed to maximize the inclusivity of search terms related to obstructive sleep apnea (OSA), cardiovascular diseases (CVDs), and relevant outcomes. Synonymous and variant expressions were utilized to ensure a thorough search strategy. Additionally, manual searches of reference lists were performed to identify additional relevant studies. Three independent reviewers screened the titles and abstracts of all identified articles, followed by a full-text review of potentially relevant articles. The search dates were from the 1st of March 2024 to the 1st of August 2024.

Keywords used in all databases include the following: OSA: (obstructive sleep apnea OR OSA) [MeSH terms]; cardiovascular diseases: (cardiovascular diseases OR heart diseases) [MeSH terms]; outcomes: (hypertension OR coronary artery disease OR congestive heart failure OR cardiac arrhythmias OR stroke OR cardiovascular events).

Data Extraction and Quality Appraisal

Quantitative and qualitative data extraction followed a rigorous process. Data were independently extracted into Microsoft Excel (Microsoft Corporation, Redmond, WA) by three reviewers and compared to ensure inter-investigator agreement. Discrepancies were resolved through discussion and consensus. General study characteristics, including author names, publication years, sample sizes, patient demographics, follow-up durations, and outcomes of interest, were recorded. Exclusion criteria comprised letters to the editor, abstracts, and studies with insufficient data. Duplicate studies with overlapping cohorts were managed to avoid overlapping bias. Language, country, patient characteristics, and underlying disease status were not restricted during the search process. Quality appraisal was performed using the Newcastle-Ottawa Scale (NOS) [15].

Statistical Analysis

A meta-analysis was conducted using Review Manager 5.4 software (Cochrane Collaboration, London, UK) to provide a comprehensive comparison of the relationship between OSA and CVDs. Relevant data extracted from selected studies were synthesized, considering various outcomes such as the risk of hypertension, coronary artery disease, congestive heart failure, cardiac arrhythmias, and cardiovascular events. Heterogeneity among studies was assessed using the I² statistic, and subgroup analyses were performed to explore potential sources of heterogeneity. Sensitivity analyses were conducted to assess the robustness of the findings. Patient-oriented data served as the basis for the primary analysis, and subgroup analyses were performed based on study design, patient characteristics, and other relevant factors.

Results

Study Selection

**Figure 1:**
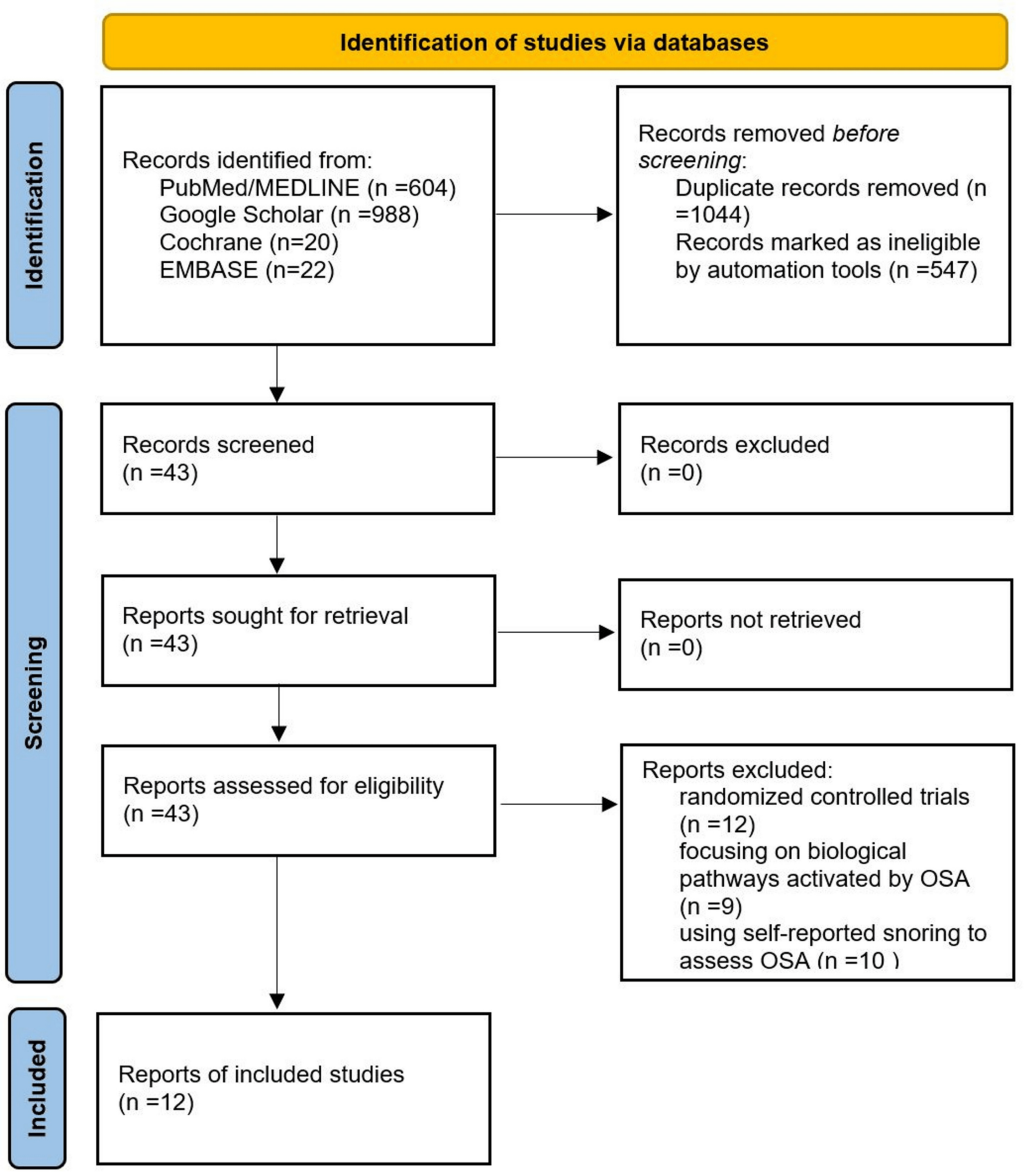
PRISMA flow diagram of the selection process. PRISMA: Preferred Reporting Items for Systematic Reviews and Meta-Analyses; OSA: obstructive sleep apnea.

The initial search yielded 1634 potentially relevant citations from the electronic reference databases. After screening the titles and abstracts, 43 articles met the inclusion criteria for detailed assessment. Among these, 31 studies were excluded for focusing on biological pathways activated by OSA, using self-reported snoring to assess OSA, or being randomized controlled trials. Ultimately, 12 studies were included in our meta-analysis. A flow diagram presenting the study selection process is shown in Figure 1.

Study Characteristics

The characteristics of the 12 identified studies are detailed in Table 1. These studies varied in sample size, follow-up duration, and outcome measures. Four studies examined the risk of overall CVDs, six assessed the risk of stroke, and two evaluated all-cause mortality.

**Table 1:** Characteristics of the included studies. ICD-9: International Classification of Diseases, Ninth Revision; DM: diabetes mellitus; SBP: systolic blood pressure; DBP: diastolic blood pressure; TC: total cholesterol; HDL-C: high-density lipoprotein cholesterol; AF: atrial fibrillation; AHI: apnea-hypopnea index; CPAP: continuous positive airway pressure; TIA: transient ischemic attack; CVD: cardiovascular disease; HTN: hypertension; PSG: polysomnogram; TC: total cholesterol; BG: blood group; SSDI: Social Security Disability Insurance; SSADMF: Social Security Administration's Death Master File.

Study ID	Recruitment	Sample size	Male Sex	Follow-up (year)	Assessment method	Outcome assessment	Outcome	Outcome events	Adjustments for confounders
Campos-Rodriguez et al. [16]	Sleep clinic, Spain	1116	0%	6	Full PSG	Medical records, computerized databases and contacting patients, certificates, relatives, or physicians, and death	Cardiovascular mortality	41	Age, BMI, DM, hypertension, and previous cardiovascular events
Gottlieb et al. [17]	Community cohorts, United States	4422	43.5	8.7	Full PSG	ICD-9 codes or medical records	Cardiovascular disease	781	Age, race, BMI, smoking, DM, SBP, DBP, TC, HDL-C, lipid-lowering medications, antihypertensive medications
Redline et al. [18]	Community cohorts	5422	45	8.7	Full PSG	Death certificates, hospital discharge information, and mailings to participants	Stroke	193	Age, BMI, race, smoking, SBP, diabetes, and antihypertensive medications
Shah et al. [19]	Sleep clinic, United States	1436	70	2.9	Full PSG	Postal questionnaire or telephone	Cardiovascular mortality	86	Age, race, sex, smoking, alcohol, BMI, AF, diabetes, hypertension, and hyperlipidemia
Punjabi et al. [20]	Community cohorts, United States	6294	46.7	8.2	Full PSG	Interviews, written questionnaires, telephone contacting participants or relatives, hospital records, community obituaries, and SSADMF	All-cause mortality	1047	Age, sex, race, BMI, SBP, DBP, smoking, prevalent hypertension, diabetes, and cardiovascular disease
Martinez-Garcia et al. [21]	Hospital center, Spain	166	59	5	Full PSG	Records from computer databases, official death certificates, contacting patients' relatives	All-cause mortality	81	Age, sex, Barthel index, AHI, and CPAP treatment groups, previous stroke or BMI, smoking, arterial hypertension, TIA, diabetes, hypercholesterolemia, atrial fibrillation, significant carotid stenosis, and fibrinogen levels
Munoz et al. [22]	Community cohort, Spain	1034	57	6	Full PSG	ICD-9 codes and medical records	Stroke	20	Sex
Arzt et al. [23]	Community cohorts, United States	1189	55	4	Full PSG	Self-report and physician diagnosis	Stroke	14	Age, sex, and BMI
Marin et al. [24]	Sleep clinic, Spain	1729	100	10.1	Full PSG	Medical records, death certificates, contacting physicians or patient's family, and SSDI	Cardiovascular mortality	68	Age, diagnostic group, presence of CVD, diabetes, hypertension, lipid disorders, "smoking, alcohol, SBP DBP, BG, TC, TG, and use of antihypertensive, lipid-lowering and antidiabetic drugs
Yaggi et al. [25]	Sleep clinic, United States	1022	71	3.4	Full PSG	Self-postal questionnaire or telephone, contacting patients or relatives	Stroke	88	Age, sex, race, smoking, alcohol, BMI, DM, hyperlipidemia, AF, and hypertension
Titova et al. [26]	Sweden	41,742	55.2	8	Full PSG	Medical records and questionnaires	Stroke	2312	Age, sex, BMI, education, smoking status, alcohol consumption, walking/biking, exercise, history of HTN, hypercholesterolemia and DM
Kojić et al. [27]	The University Clinical Center in Tuzla, Bosnia and Herzegovina	110	59	1	Full PSG	Medical records, questionnaires, and interviews	Stroke	19	-

Quality Assessment of Included Studies

The NOS was utilized to assess the quality of the studies included in this analysis, as shown in Table 2. The majority of the studies exhibited a low risk of bias. For instance, studies by Titova et al., Gottlieb et al., and Redline et al. demonstrated strong methodology with proper selection of cohorts, adequate follow-up periods, and comprehensive outcome assessments [17,18,26]. These studies consistently showed high comparability and representativeness of the exposed cohorts, contributing to their low risk of bias rating. In contrast, the study by Kojić et al. was identified as having a high risk of bias due to inadequate follow-up and incomplete assessment of outcomes [27]. The overall quality assessment indicates that while most studies were robust, a few exhibited methodological weaknesses that could affect their findings.

**Table 2:** Newcastle-Ottawa Scale (NOS) results of the included studies. * Presence. * Strong presence.

Study	Representativeness of the exposed cohort	Selection of the non-exposed cohort	Ascertainment of exposure	Demonstration that outcome of interest was not present at the start of the study	Comparability of cohorts on the basis of the design or analysis	Assessment of outcome	Was follow-up long enough for outcomes to occur?	Adequacy of follow-up of cohorts	Final judgment
Campos-Rodriguez et al. [16]	*	*		*	**	*	*	*	Low risk of bias
Gottlieb et al. [17]	*	*	*	*		*			High risk of bias
Redline et al. [18]	*		*	*	**	*	*	*	Low risk of bias
Shah et al. [19]	*	*	*	*	**	*	*	*	Low risk of bias
Punjabi et al. [20]	*		*	*	**	*	*	*	Low risk of bias
Martinez-Garcia et al. [21]	*		*	*	**	*	*	*	Low risk of bias
Munoz et al. [22]	*	*	*	*	**	*	*		Low risk of bias
Arzt et al. [23]	*		*	*	**	*	*	*	Low risk of bias
Marin et al. [24]	*		*		**	*	*	*	Low risk of bias
Yaggi et al. [25]	*		*	*	**	*	*	*	Low risk of bias
Titova et al. [26]		*	*	*	*	*	*		Low risk of bias
Kojić et al. [27]	*		*	*	**	*	*	*	Low risk of bias

Meta-Analysis Results

**Figure 2:**
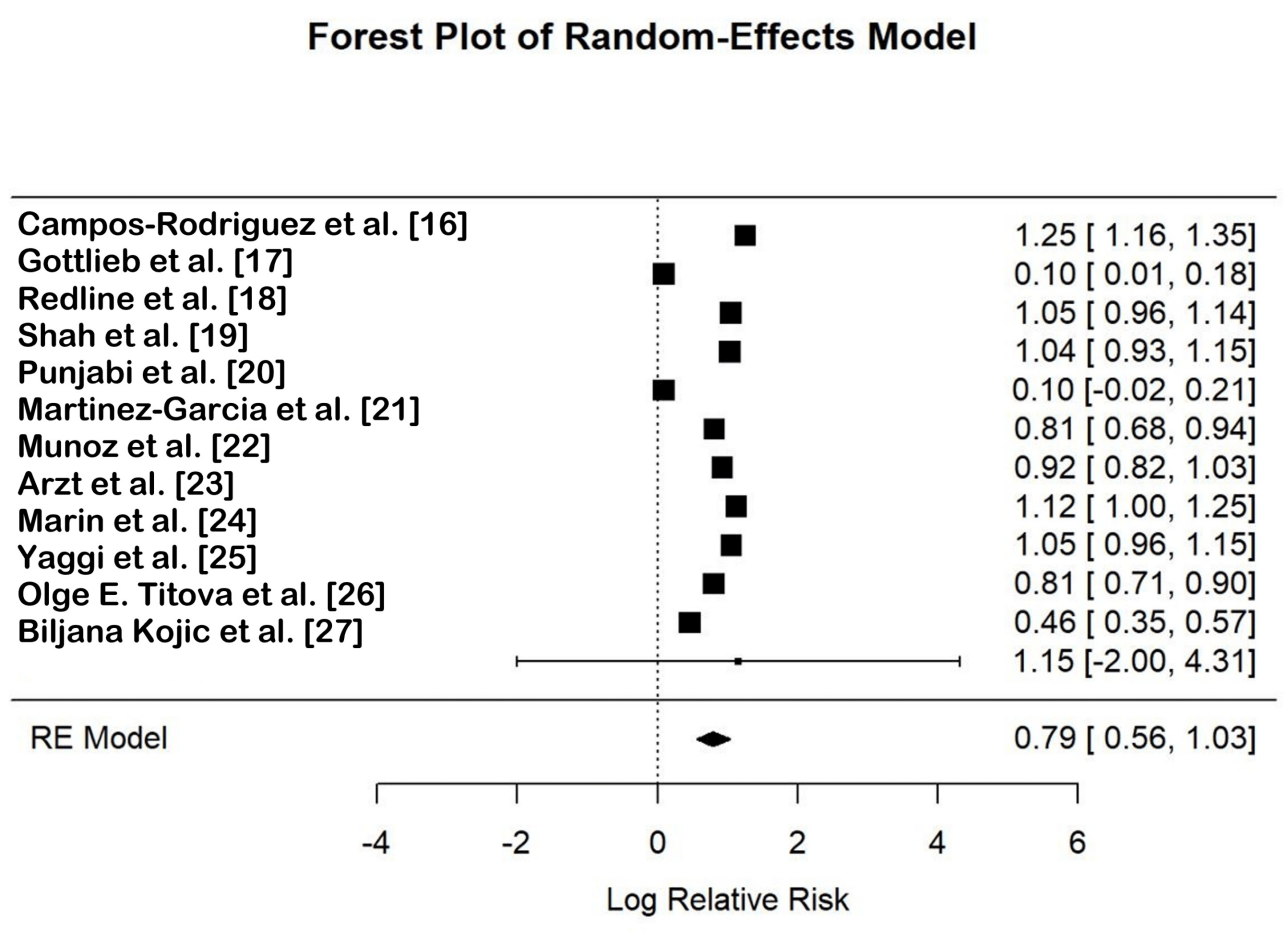
Forest plot of random-effects model. RE: random effects.

The forest plot (Figure 2) summarizes the log relative risks (RRs) and 95% confidence intervals (CI) for each study, as well as the overall effect estimate from the random-effects model. The pooled RR from the random-effects model was 0.79 (95% CI: 0.56, 1.03), indicating a non-significant reduction in cardiovascular risk associated with OSA. The individual study estimates varied, with some studies showing increased risk and others showing decreased risk.

**Figure 3:**
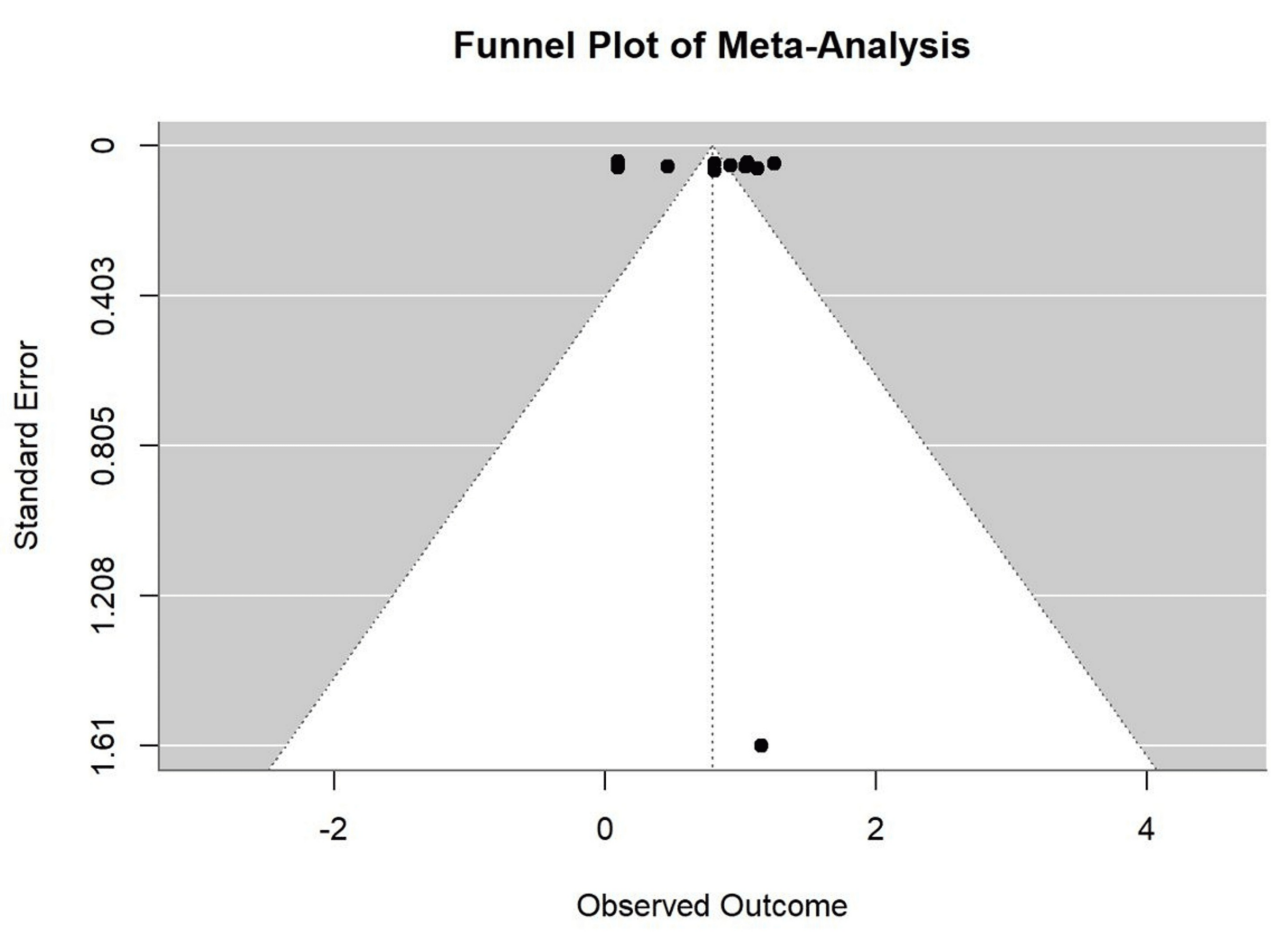
Funnel plot of the studies included in the meta-analysis.

The funnel plot (Figure 3) was used to assess the potential for publication bias in the meta-analysis. Ideally, in the absence of bias, the plot should resemble a symmetric inverted funnel. In our analysis, the plot shows some degree of asymmetry with several studies falling outside the funnel. This suggests the possibility of heterogeneity among the included studies.

Subgroup Analyses

The analysis of cardiovascular mortality showed several findings, including the following: Campos-Rodriguez et al. reported a RR of 1.25 (95% CI: 1.16, 1.35), indicating a significant increase in cardiovascular mortality among individuals with OSA [16]. Shah et al. found an RR of 1.04 (95% CI: 0.93, 1.15), showing a non-significant increase in risk [19]. In contrast, Munoz et al. observed an RR of 0.81 (95% CI: 0.68, 0.94), suggesting a protective effect, while Marin et al. reported an RR of 1.12 (95% CI: 1.00, 1.25), indicating a significant increase in risk [22,24].

Regarding the risk of stroke, Redline et al. found an RR of 1.05 (95% CI: 0.96, 1.14), showing a non-significant increase in stroke risk [18]. Martinez-Garcia et al. observed an RR of 0.10 (95% CI: -0.02, 0.21), indicating a non-significant reduction in risk [21]. Yaggi et al. reported an RR of 1.05 (95% CI: 0.96, 1.15), showing a non-significant increase in risk, whereas Titova et al. observed an RR of 0.46 (95% CI: 0.35, 0.57), indicating a significant reduction in stroke risk [25,26].

For all-cause mortality, Punjabi et al. reported an RR of 0.10 (95% CI: -0.02, 0.21), indicating a non-significant reduction in all-cause mortality [20]. Martinez-Garcia et al. found an RR of 0.81 (95% CI: 0.68, 0.94), suggesting a significant reduction in risk [21]. Arzt et al. observed an RR of 0.92 (95% CI: 0.82, 1.03), showing a non-significant reduction in risk [23].

Discussion

This meta-analysis investigated the relationship between OSA and CVDs, synthesizing data from 12 studies. The pooled RR was 0.79 (95% CI: 0.56-1.03), indicating a non-significant reduction in cardiovascular risk associated with OSA. However, the results were heterogeneous, with some studies showing increased risk and others showing decreased risk.

The precise mechanisms contributing to the heightened risk of CVD in patients with OSA remain somewhat uncertain, and the existing data are conflicted. Data from the present meta-analysis somewhat support the notion that OSA is an important, emerging risk factor for CVD. Despite accumulative evidence supporting a positive relationship, a causal association between OSA and CVD is, however, not established. Our meta-analysis showed a non-significant reduction in the CVD risk associated with OSA. In contrast, Dong et al., in their meta-analysis which included 17 studies (nine reporting on total CVD, seven on fatal or non-fatal coronary heart disease (CHD), and 10 on fatal or non-fatal stroke), found that individuals with moderate to severe OSA had significantly higher risks of cardiovascular events [28].

The pooled RRs were 2.48 for total CVD, 1.37 for CHD, and 2.02 for stroke compared to the reference group. These findings remained consistent across sensitivity analyses. Their study concluded that moderate to severe OSA significantly increases cardiovascular risk, particularly the risk of stroke.

Similarly, Wang et al. conducted a meta-analysis using generalized least squares regression models to estimate the dose-response relationship and performed heterogeneity, subgroup, and sensitivity analyses. Including 12 prospective cohort studies with 25,760 participants, they found that individuals with severe OSA had significantly higher risks of CVD, stroke, and all-cause mortality. The overall combined RRs were 1.79 for CVD, 1.21 for CHD, 2.15 for stroke, and 1.92 for all-cause mortality [29]. They also observed a positive association with CVD for moderate OSA but not for mild OSA. The authors concluded that severe OSA significantly increases the risk of CVD, stroke, and all-cause mortality. Both studies suggest that the risk of CVD is dependent on the severity of OSA. This is further supported by the findings of the meta-analysis conducted by Salari et al. [30]. However, Zhang et al. reported that even mild OSA can lead to an increased risk, suggesting that it may not be a dose-dependent response [31].

In contrast to these findings, our meta-analysis did not show a statistically significant reduction in cardiovascular risk associated with OSA, which may be due to heterogeneity among studies, differences in study design, or variations in the populations studied. CVD outcomes are heavily influenced by socioeconomic status (SES) and race. Non-Hispanic Black and Hispanic populations experience higher rates of hypertension, obesity, and diabetes, compounded by systemic barriers like lower income and poor healthcare access. Addressing these disparities requires targeted interventions that factor in SES and race to reduce cardiovascular morbidity and mortality [32]. Despite this, our results support the notion that OSA is an emerging risk factor for CVD. The clinical implications of such findings are significant, suggesting that routine screening for OSA in patients with or at risk for CVDs could be beneficial. Given the observed association between OSA and increased cardiovascular risk [33], incorporating sleep assessments into standard cardiovascular care could lead to earlier diagnosis and intervention. This is particularly important because OSA often remains undiagnosed, especially among patients with pre-existing cardiovascular conditions, despite its high prevalence and potential for serious health consequences.

Early diagnosis and management of OSA, particularly through the use of CPAP therapy, could potentially reduce the incidence of cardiovascular events. CPAP therapy, which is currently the standard treatment for OSA, works by maintaining airway patency during sleep, thereby reducing the frequency of apneic episodes and the associated intermittent hypoxemia. This reduction in hypoxemia may mitigate the downstream effects of sympathetic activation, systemic inflammation, and endothelial dysfunction - key mechanisms linking OSA to CVDs [34].

Our results suggest that CPAP therapy could play a crucial role in not only improving sleep quality and reducing daytime symptoms but also in lowering the cardiovascular risk associated with OSA. However, the extent of this benefit remains a topic of debate, as some studies have shown mixed results regarding CPAP's effectiveness in reducing cardiovascular events [35]. Despite this, the potential for CPAP therapy to positively impact cardiovascular outcomes warrants its consideration as a central component of managing patients with both OSA and CVD [36]. In light of these findings, there is a strong case for integrating sleep assessments into the routine evaluation of patients at risk for CVDs. This approach could lead to more comprehensive care strategies that address both sleep disorders and cardiovascular risk factors, ultimately improving patient outcomes. Additionally, these findings highlight the need for ongoing research to further clarify the impact of OSA treatment on cardiovascular health and to refine guidelines for the screening and management of OSA in cardiovascular populations.

This meta-analysis has several important limitations. Publication bias remains a concern, as studies with significant findings are more likely to be included, despite attempts to mitigate this through comprehensive searches and funnel plot analysis. The observational nature of most studies limits establishing a direct causal link between OSA and CVDs. Additionally, reliance on self-reported data may introduce reporting bias, affecting accuracy. Variations in study designs, diagnostic criteria, and populations, as well as uncontrolled confounders, further limit the generalizability and consistency of the findings.

Based on the findings and limitations of this study, several recommendations can be made for future research and clinical practice. First, there is a need for more high-quality, prospective studies that use standardized definitions and diagnostic criteria for OSA and cardiovascular outcomes to reduce heterogeneity and improve comparability across studies. Additionally, exploring the potential causal mechanisms linking OSA to CVDs through well-designed randomized controlled trials could provide more definitive evidence on the impact of OSA treatment, such as CPAP therapy, on reducing cardiovascular risk. Clinically, it is recommended that healthcare providers consider routine screening for OSA in patients with or at risk for CVDs, as early diagnosis and management may mitigate the associated cardiovascular risks. Integrating sleep assessments into standard cardiovascular care could lead to more comprehensive management strategies, ultimately improving patient outcomes. For example, implementing questionnaires such as the STOP-Bang or Epworth Sleepiness Scale can help identify at-risk patients. Additionally, referring patients with symptoms of sleep issues for polysomnography (sleep studies) or home sleep apnea testing would allow early detection and treatment. Coordinating care with sleep specialists and integrating CPAP therapy for OSA could further improve cardiovascular outcomes.

## Conclusions

This meta-analysis highlights the complex relationship between OSA and CVDs, emphasizing the need for further research to clarify the underlying mechanisms and establish a causal link. While the findings suggest a potential association between OSA and increased cardiovascular risk, the evidence remains inconclusive due to variability in study design and outcomes. Nevertheless, the study underscores the importance of early detection and management of OSA, particularly in individuals with existing cardiovascular risk factors. Integrating sleep assessments into routine cardiovascular care could be a valuable step toward reducing the burden of CVDs.
